# The role of gut microorganisms and metabolites in intracerebral hemorrhagic stroke: a comprehensive review

**DOI:** 10.3389/fnins.2024.1346184

**Published:** 2024-02-21

**Authors:** Xin Wen, Hao Dong, Wei Zou

**Affiliations:** ^1^The First Clinical Medical College, Heilongjiang University Of Chinese Medicine, Harbin, China; ^2^The First Affiliated Hospital of Heilongjiang University of Chinese Medicine, Harbin, China

**Keywords:** intracerebral hemorrhage, microbiota-gut-brain axis, short-chain fatty acids, trimethylamine N-oxide, ghrelin, NLRP3 inflammasome

## Abstract

Intracerebral hemorrhagic stroke, characterized by acute hemorrhage in the brain, has a significant clinical prevalence and poses a substantial threat to individuals’ well-being and productivity. Recent research has elucidated the role of gut microorganisms and their metabolites in influencing brain function through the microbiota-gut-brain axis (MGBA). This article provides a comprehensive review of the current literature on the common metabolites, short-chain fatty acids (SCFAs) and trimethylamine-N-oxide (TMAO), produced by gut microbiota. These metabolites have demonstrated the potential to traverse the blood–brain barrier (BBB) and directly impact brain tissue. Additionally, these compounds have the potential to modulate the parasympathetic nervous system, thereby facilitating the release of pertinent substances, impeding the buildup of inflammatory agents within the brain, and manifesting anti-inflammatory properties. Furthermore, this scholarly analysis delves into the existing dearth of investigations concerning the influence of gut microorganisms and their metabolites on cerebral functions, while also highlighting prospective avenues for future research.

## 1 Introduction

Intracerebral hemorrhage (ICH) has a global impact on individuals’ productivity and overall well-being. This condition, classified as a cerebrovascular disease, arises from the non-traumatic rupture of cerebral parenchymal vessels, leading to impaired brain function. The clinical manifestations of ICH exhibit considerable diversity, with varying symptoms depending on the location and extent of the bleeding. The detrimental effects of ICH on brain tissue can be categorized primarily into physical and chemical damage. Physical damage primarily arises from hematoma compression resulting from the site of bleeding, while chemical damage is attributed to the infiltration of diverse substances from the bloodstream into the brain. The underlying pathological mechanism of ICH is intricate, and the precise molecular mechanism remains to be elucidated.

The microbiota-gut-brain axis (MGBA), also known as the brain-gut axis, is a neural-endocrine network system comprising the brain, gut, and gut microbiota. Within the human gut, a diverse range of microorganisms such as bacteria, viruses, and parasites coexist, forming a symbiotic system through prolonged interaction with humans. Recent research has demonstrated the extensive interconnections between the MGBA and the central nervous system (CNS; Ma et al., 2019). Damage to the CNS has been observed to coincide with alterations in gut microbiota, while changes in the abundance of gut microbiota have also been found to impact the CNS. The involvement of gut microbiota in the pathogenesis and progression of diseases such as Alzheimer’s disease (Liu et al., 2020), Parkinson’s disease (Chandra et al., 2017), and ischemic stroke (Zhang et al., 2023) have been established, although limited research exists regarding its role in cerebral hemorrhage. This review critically examines previous literature pertaining to the manipulation of the MGBA for the treatment of ICH, and explores potential proteins that hold promise as therapeutic targets, thereby offering novel avenues for the future management of ICH.

## 2 The function of MGBA in ICH

### 2.1 The mechanism of intestinal microbiota’s effect on brain tissue after ICH

The CNS assumes a crucial role in the regulation of intestinal homeostasis and gastrointestinal function, while reciprocal regulation of the CNS by gut microbes is also observed. This concept serves as the foundation for the MGBA. Traditionally, the transmission of signals relied solely on unmyelinated vagal and sympathetic afferent fibers. However, recent microbiological investigations have revealed that the gut microbiota and its metabolites serve as an additional significant source of signal transmission. Consequently, microbes possess the ability to impact various aspects of brain structure and function through the neuro-immune system and neuro-endocrine system (Martin et al., 2018; Osadchiy et al., 2019).

Based on the findings of Mulak and Bonaz (2004), the regulation of intestinal nerves is influenced by various factors, including the enteric nervous system (ENS), the vagus nerve, the immune system, and the presence of intestinal microorganisms and their metabolites. The first level of regulation involves the ENS, where enteric motor neurons and sensory neurons are interconnected, enabling autonomous organization and processing of information. The second level of regulation occurs at the prevertebral ganglion, which receives and transmits neural signals from both the CNS and the ENS. The third tier encompasses the CNS, which assimilates and analyzes both external and internal stimuli, subsequently transmitting regulatory information to the ENS and effector organs via either the autonomic nervous system or the neuroendocrine system. The fourth tier pertains to the regulation of the superior neural centers, wherein neural signals originating from the cerebral cortex and subcortical regions converge within the basal ganglia, ultimately emitting signals to govern bodily functions (Zhu et al., 2017).

There exist numerous cellular targets of intestinal neurons, encompassing interstitial cells of Cajal (ICCs) as well as immune cells, including mast cells and T cells (Ward, 2000). ICCs are widely acknowledged to assume a crucial function in neural transmission and possess the capability to convey signals to the brain via the vagus nerve (Choe et al., 2021). Additionally, mast cells constitute another significant cell type involved in MGBA. The ENS has the capacity to convey signals to the CNS via sympathetic and parasympathetic nerves (Costa et al., 2000; Holzer et al., 2001). Anatomical investigations have demonstrated that both sympathetic and parasympathetic nerves generate branches upon entering the intestinal wall, establishing connections not only with intestinal neurons but also with immune cells, including mast cells. Activation of the vagus nerve can modulate mast cell activity, consequently influencing brain function. This structural arrangement potentially serves as a foundational element underlying the manifestation of the MGBA (Costa et al., 2000; Stead et al., 2006).

Following the incidence of ICH, the human body undergoes stress-induced stimulation, leading to diminished intestinal perfusion, intestinal mucosa edema, impairment of intestinal barrier function, heightened intestinal permeability, and activation of sympathetic nerves. These factors contribute to the development of intestinal epithelial dysfunction and dysbiosis of intestinal microbiota, consequently facilitating the release of 5-hydroxytryptamine (5-HT) from intestinal chromaffin cells (Richards et al., 2017). Research has demonstrated that intestinal microbial communities possess the ability to regulate the biosynthesis of 5-HT, facilitate the maturation of the ENS, and consequently exert an influence on both brain development and function (Lyte, 2014). Inflammatory processes have the potential to induce heightened cytokine activity, enhanced motility, and the release of neurotransmitters, including 5-HT, thereby interfering with the communication between the CNS and ENS (Ringel et al., 2001). Due to the inability of intestinal 5-HT to cross the blood–brain barrier (BBB), the central and peripheral systems can be considered as two independent systems. However, recent studies have indicated that the presence of 5-HT in peripheral blood can potentially influence the CNS by modulating the expression of tryptophan (Agus et al., 2018). Given its crucial role as a mediator in the development and functioning of both the ENS and CNS, 5-HT may hold significant importance in facilitating communication within the MGBA (Huang et al., 2023).

### 2.2 Effect of brain tissue injury after ICH on MGBA

According to recent research, ICH has been found to be associated with alterations in the composition of the gut microbiota. Li W. et al. (2022) demonstrated significant changes in the abundance of *Firmicutes*, *Proteobacteria*, and *Verrucomicrobia* in the gut microbiota following the occurrence of ICH, as compared to a healthy gut microbiota. At the taxonomic level of genus, a notable decrease in the abundance of *Bacteroides, Blautia, Faecalibacterium, Subdoligranulum, Bifidobacterium, Agathobacter*, and *Romboutsia* was observed, whereas a significant increase in the abundance of *Escherichia-Shigella*, *Akkermansia*, *Lactobacillus*, and *Lachnoclostridium* was evident. These findings suggest that ICH has the potential to induce alterations in the gut microbiota composition. A study has demonstrated that alterations in the gut microbiota composition can result in impairment of the intestinal barrier function (Ferguson et al., 2019). However, the potential of gut microbiota composition changes following ICH to induce subsequent damage to the intestinal or brain tissues requires further investigation.

## 3 Multiple factors that may be used to treat ICH through MGBA

### 3.1 NLRP3 inflammasome

Prolonged exposure of the human body to detrimental stimuli can result in the activation of the immune system, leading to the release of inflammatory cells and the initiation of inflammatory responses. The regulation of inflammasome assembly and signaling plays a vital role in facilitating the immune system’s ability to exert anti-inflammatory effects. The formation of each inflammasome is determined by pattern-recognition receptors (PRRs), which detect endogenous danger signals generated within the body or pathogen-associated molecular patterns (PAMPs) present in cells. Nod-like receptor 3 (NLRP3) is a prototypical PRR that, in conjunction with apoptosis-associated speck-like protein containing a caspase-recruitment domain (ASC) and pro-caspase-1, constitutes the NLRP3 inflammasome. Upon stimulation by extracellular inflammatory stimuli, the NLRP3 inflammasome becomes activated, leading to the activation of pro-caspase-1 into caspase-1 and the subsequent release of inflammatory mediators such as IL-1β and IL-18. Additionally, Gasdermin D (GSDMD) is dissociated and interacts with the cell membrane to form pores, thereby initiating pyroptosis (Zhang et al., 2022).

Recent research has demonstrated that the activation of the NLRP3 inflammasome exerts an influence on the composition of gut microorganisms (Zhang et al., 2019). Conversely, alterations in the gut microbiota can also trigger the activation of the inflammasome and enhance the expression of inflammatory cells, thereby exacerbating neuroinflammation (Tirelle et al., 2021). Following the incidence of ICH, there is a disruption in the composition and structure of the gut microbiota, characterized by a reduction in the proportion of beneficial bacteria and an elevation in the proportion of detrimental bacteria. The underlying mechanism for this alteration in the gut microbiota, as a consequence of brain injury, can potentially be elucidated through the activation of the NLRP3 inflammasome. The correlation between the dysregulation of gut microbiota and the NLRP3 inflammasome has been documented in various CNS disorders. Shen et al. (2020) conducted a transplantation of fecal matter from individuals diagnosed with Alzheimer’s disease into the intestines of APP/PS1 double transgenic mice. The researchers observed the activation of the NLRP3 inflammasome in the mice, along with an increase in proinflammatory factors, exacerbation of neuroinflammation, and cognitive impairment. Zhang et al. (2019) conducted fecal transplantation experiments involving depression and anxiety model rats and wild-type mice, as well as NLRP3 gene knockout mice. The findings revealed that the transplanted wild-type mice displayed depression and anxiety-like behaviors, whereas the NLRP3 gene knockout mice exhibited milder manifestations of these behaviors compared to the wild-type mice. These experimental results suggest that alterations in the gut microbiota have the potential to exacerbate brain lesions, with NLRP3 potentially serving as a mediator in this process.

Following the incidence of ICH, activated microglia release a multitude of inflammatory factors, including IL-1β (Shen et al., 2017), which may further aggravate the secondary damage to brain tissue subsequent to ICH. The involvement of the NLRP3 inflammasome in the release of inflammatory factors by microglia has been established (Wang D. et al., 2017). Consequently, regulating the expression of the NLRP3 inflammasome assumes paramount importance in the prevention of secondary injury to brain tissue. The precise mechanism by which the NLRP3 inflammasome contributes to brain tissue injury remains incompletely understood in current research. However, previous studies have demonstrated that the NLRP3 inflammasome plays a significant role in the pathogenesis of brain tissue injury following ICH by facilitating the release of various inflammatory factors, particularly IL-1β (Ma et al., 2014; Ren et al., 2018). In rats subjected to ICH, IL-1β and neutrophils can be observed in the vicinity of the hematoma after 3 h. Notably, when the NLRP3 gene is knocked out in ICH rats, the levels of IL-1β and neutrophils surrounding the hematoma decrease, leading to a reduction in brain edema severity. The utilization of siRNA to silence the NLRP3 inflammasome has been demonstrated to yield comparable outcomes (Ma et al., 2014; Chen et al., 2020), thereby suggesting the involvement of both NLRP3 and the NLRP3 inflammasome in the amplification of IL-1β inflammatory signaling subsequent to ICH, ultimately leading to brain tissue damage. Suppression of NLRP3 inflammasome expression not only mitigates brain inflammation but also safeguards the CNS. MCC950 serves as a discerning inhibitor of the NLRP3 inflammasome (Coll et al., 2019). Xiao et al. (2022) demonstrated that following ICH, there was an alteration in the composition of the intestinal microbial community in rats, characterized by a substantial increase in the abundance of *Helicobocton Pyloni*. Subsequent administration of MCC950 in ICH mice resulted in a shift in the composition of the intestinal microbial community, marked by a significant increase in the populations of *Bifidobacterium* and *Bacteroides*. These particular strains are commonly acknowledged as probiotics, known to aid in the regulation of gastrointestinal dysfunction. The alterations in the immunostaining intensity of myelin basic protein (MBP) and neurofilament 200 (NF200) have the potential to provide insights into the general state of the CNS to some degree (Wang R. et al., 2017). Following administration of MCC950, the MBP/NF200 ratio decreased in ICH mice, suggesting that suppression of the NLRP3 inflammasome expression can not only enhance the composition of the gut microbiota and augment the prevalence of beneficial bacteria, but also confer protection to the CNS. The study conducted by Zhao et al. (2022) demonstrated that modulating the composition of the intestinal microbiota and preserving the integrity of the intestinal barrier can effectively suppress the activation of the NLRP3 inflammasome and the expression of associated inflammatory factors. These findings offer potential for utilizing MGBA to enhance the intestinal microbiota as a viable approach to treating ICH, and hold considerable importance in the prevention and management of secondary brain damage following ICH.

### 3.2 Ghrelin

#### 3.2.1 Protective effect of ghrelin on brain tissue after ICH

Ghrelin, a multifunctional brain gut peptide primarily secreted in the stomach, is also highly expressed in the pituitary and hypothalamus regions of the CNS (Ghelardoni et al., 2006). Additionally, ghrelin in peripheral blood has the ability to exert its functions in the CNS by crossing the BBB (Rhea et al., 2018). Recent research has demonstrated the remarkable anti-inflammatory, antioxidant, anti-apoptotic, and other effects of ghrelin in the CNS, leading to its widespread recognition (Stempniewicz et al., 2019). However, despite these notable effects, the precise mechanism of action of ghrelin remains unclear. There exist multiple pathways responsible for regulating pro-inflammatory signal transduction, one of which is the NF-κB pathway. Following ICH, numerous inflammatory factors, such as TNF-α and IL-1β, are recruited to brain tissue (Shen et al., 2017). The expression of TNF-α and IL-1β facilitates the activation of NF-κB, which in turn enhances the gene transcription of TNF-α and IL-1β, thereby amplifying their expression. Consequently, this perpetuates a vicious cycle by further activating additional NF-κB. Recent research has demonstrated that ghrelin possesses the ability to impede the activation of NLRP3 inflammasome by suppressing the TNF-α-induced NF-κB pathway and the subsequent recruitment of inflammatory mediators. Consequently, this mechanism safeguards the CNS and effectively accomplishes the objectives of mitigating inflammation and pyroptosis (Li et al., 2004; Liu F. et al., 2019; Cheng et al., 2020). Research has demonstrated that inflammation plays a substantial role in the secondary injury caused by ICH, with activated microglia releasing inflammatory factors that worsen neural damage in the CNS (Shen et al., 2017). Given ghrelin’s remarkable anti-inflammatory capacity, it can effectively impede the activation of NF-κB and NLRP3 inflammasome following ICH, consequently restraining subsequent neuroinflammation and safeguarding the CNS (Cheng et al., 2020).

Furthermore, it is noteworthy to acknowledge the antioxidant capability of ghrelin, in addition to its remarkable anti-inflammatory efficacy (Stempniewicz et al., 2019). According to a study, ICH leads to the occurrence of oxidative stress (OS) and lipid peroxidation processes in brain tissue (Duan et al., 2016; Bai et al., 2020). The presence of OS is considered a significant contributor to the subsequent damage observed in secondary brain tissue following ICH (Shao et al., 2022). Consequently, it is imperative to intervene and inhibit this process in order to safeguard the integrity of brain tissue. Obay et al. (2008) discovered that ghrelin exhibited a dose-dependent preventive effect on the reduction of glutathione (GSH) levels and antioxidant enzyme activity in mouse brain tissue following intraperitoneal injection of ghrelin pretreated epilepsy model mice. Additionally, the researchers observed that ghrelin demonstrated robust antioxidant capabilities in brain tissue. Given that adult growth hormone deficiency can result in oxidative stress (Kokoszko et al., 2008), and ghrelin possesses the ability to stimulate growth hormone secretion (Fukumori et al., 2012), further investigation is required to determine whether the antioxidant effect of ghrelin is direct or mediated through growth hormone stimulation. A study has demonstrated an association between growth hormone and 30-day mortality following ICH, as well as a deterioration in prognosis after 90 days (Zweifel et al., 2011). However, the precise mechanism by which this association occurs remains uncertain. Therefore, it is worthwhile to conduct further research to investigate whether ghrelin can impact the prognosis of ICH patients through the regulation of growth hormone. Cheng et al. (2020) conducted a study in which ghrelin was administered to mice with ICH, revealing that ghrelin possesses the ability to inhibit OS through the activation of the Nrf2/ARE antioxidant pathway. The administration of ghrelin resulted in an upregulation of total Nrf2 expression at both mRNA and protein levels, enhanced binding activity of Nrf2 DNA post-ICH, and facilitated the nuclear translocation of Nrf2, thereby activating downstream antioxidant genes (e.g., GCLC, GCLM, etc.) to exert antioxidant effects. Although this study demonstrates the antioxidant role of ghrelin through the activation of the Nrf2/ARE pathway, the precise molecular mechanism underlying this phenomenon remains to be elucidated, necessitating further comprehensive investigation.

Ghrelin exhibits anti-apoptotic effects within the CNS (Chung et al., 2007). Bax and Bcl-2 are apoptosis-associated proteins that are members of the Bcl-2 family. Specifically, Bax has the ability to form dimers, thereby enhancing cell membrane permeability, whereas Bcl-2 can interact with Bax to diminish cell membrane permeability. The expression ratio of Bcl-2 and Bax can serve as an indicator of cellular resistance to apoptosis to a certain degree. An increase in the Bax/Bcl-2 ratio promotes apoptosis, while a decrease in the ratio inhibits apoptosis (Adams and Cory, 1998). Research has demonstrated that ghrelin in the CNS can activate the PI3k/Akt and MAPK pathways in rats, leading to a reduction in Bax protein expression and an increase in Bcl-2 protein expression. This subsequently lowers the Bax/Bcl-2 ratio, inhibits Caspase-3 activation, and ultimately achieves an anti-apoptotic effect (Chung et al., 2008; Bai et al., 2022). Apoptosis emerges as the primary mechanism of cellular demise in the vicinity of hematoma within the brain subsequent to ICH (Qureshi et al., 2003). However, the existing body of research pertaining to the therapeutic potential of ghrelin in mitigating apoptosis following ICH remains considerably restricted, thereby warranting further investigation as a prospective avenue for future studies.

#### 3.2.2 Protective effect of ghrelin on intestine after ICH

ICH has been found to result in impaired intestinal barrier function, heightened intestinal permeability, and modified gut microbiota composition (Yu et al., 2021). Conversely, alterations in the composition of intestinal microbes have also been observed to contribute to impaired intestinal barrier function and increased intestinal permeability (Ferguson et al., 2019). Research has indicated that heightened intestinal permeability can lead to an elevated concentration of endotoxins in the peripheral blood, with the potential for these endotoxins to traverse the BBB and impact the CNS, thereby exacerbating brain inflammation (Silverman et al., 2015; Liu Z. et al., 2019). Ghrelin has the potential to not only address CNS injury following ICH, but also to alleviate intestinal barrier dysfunction subsequent to ICH. The integrity of the intestinal barrier is primarily maintained by tight junctions formed between intestinal epithelial cells, which consist of various molecules such as claudin-5 (CLDN-5) and zonula occludens-1 (ZO-1; Watari et al., 2016; Zhu H. et al., 2020). Cheng et al. (2016) employed ghrelin as a therapeutic intervention for rats with ICH and observed that ghrelin exhibited the ability to enhance the expression of CLDN-5 protein and ZO-1 protein, thereby suggesting a beneficial impact on the restoration of intestinal barrier function in ICH rats. Another plausible mechanism through which ghrelin may facilitate the repair of the intestinal barrier in rats is by inhibiting NF-κB. Cheng et al. (2015) employed ghrelin as a therapeutic intervention for colitis-afflicted rats. This intervention resulted in an amelioration of the intestinal barrier function, a decrease in the levels of the inflammatory factor NF-κB, and an enhancement of the tight junction integrity within the intestinal barrier. These findings suggest that ghrelin may safeguard the integrity of the intestinal barrier by suppressing the expression of NF-κB. However, additional investigation is warranted to elucidate the precise mechanism of action and to determine whether this mode of action is relevant in the context of intestinal damage following ICH.

### 3.3 Ghrelin and MGBA

The Growth hormone secretagogue receptor (GHSR) serves as an endogenous ligand of ghrelin, enabling it to bind to GHSR and elicit its effects (Kojima et al., 1999). GHSR receptors are present in the myenteric plexus, and GHSR mRNA is expressed in both the intestinal wall and myenteric plexus (Xu et al., 2005). Additional investigations have demonstrated the expression of ghrelin in the myenteric plexus (Takeshita et al., 2006). Consequently, it is hypothesized that ghrelin may activate peripheral receptors of the ENS through its interaction with GHSR. Peeters (2005) provided a comprehensive synthesis of prior experimental findings, positing the existence of two distinct mechanisms within the ENS: the local pathway and the central pathway. In the absence of any disruptions, the central pathway exclusively governs the ENS function in healthy rats. However, when the central pathway becomes inaccessible, as in the case of vagotomy, ghrelin assumes a functional role by acting through the myenteric plexus. This phenomenon potentially elucidates the underlying mechanism through which ghrelin exerts its protective influence on the intestines. Ghrelin exhibits dual pathway action, with the activation of the central pathway leading to an upregulation of ghrelin expression. Research has demonstrated that chemical genetic activation of the vagus nerve results in increased plasma ghrelin expression. Conversely, when the vagus nerve is impaired, there is a decrease in both total ghrelin and active ghrelin expression in plasma. Additionally, the investigation revealed the presence of a direct conduit connecting the gastric fundus to the vagus nerve (Liu et al., 2022). Consequently, this study posits that the vagus nerve potentially exerts direct influence on the gastric fundus, thereby facilitating the upregulation of ghrelin expression and subsequently elevating ghrelin levels in the bloodstream.

Intestinal microorganisms play a crucial role in the functioning of the MGBA. Recent research has demonstrated that these microorganisms have the potential to modulate the expression of ghrelin. Specifically, *Bacteroides*, *Escherichia*, and *Blautia* exhibit an inverse relationship with ghrelin expression, whereas *Akkermansia* demonstrates a positive association with ghrelin expression (Liu et al., 2017). Short-chain fatty acids (SCFAs), such as acetate, propionic acid, and butyrate, are the byproducts of dietary fiber metabolism by intestinal microorganisms. Perry et al. (2016) demonstrated that elevated levels of acetate can activate the parasympathetic nervous system, thereby facilitating the release of ghrelin. This finding implies that the gut microbiota can influence the CNS through the conversion of dietary components into SCFAs, which in turn stimulate the parasympathetic nervous system and enhance ghrelin secretion.

## 4 Intestinal microbial metabolites and ICH

The composition of intestinal microorganisms primarily encompasses advantageous, detrimental, and neutral microbial assemblages, accompanied by a diverse range of metabolites such as trimethylamine (TMA)/trimethylamine N-oxide (TMAO), SCFAs, and others. TMA is derived from *Lachnospiraceae*, *Ruminococcaceae*, *Lactobacillus*, and *Blautia* bacteria (Cai et al., 2022), whereas SCFAs are metabolized by *L. Saccharolyticum*, *Escherichia*, *Klebsiella*, and *Shigella* bacteria (Li et al., 2023). Recent research has demonstrated the potential impact of intestinal microbial metabolites on various diseases, including cardiovascular and cerebrovascular ailments (Katsimichas et al., 2019).

Hypertension and atherosclerosis are significant risk factors that impact the occurrence and prognosis of ICH (An et al., 2017). Timely intervention and mitigation of risk factors hold positive implications for the management of ICH. Recent research has revealed a correlation between hypertension and the presence of gut microorganisms, as well as their metabolites known as SCFAs.

In individuals with hypertension, there is a reduced prevalence of *Ruminococcaceae* spp. or *Roseburia* spp. in the intestinal tract (Sun et al., 2019), whereas there is an increased prevalence of *Klebsiella* spp. and *Streptococcaceae* spp. (Verhaar et al., 2020). The correlation analysis between blood pressure and SCFAs revealed a positive association between blood pressure and SCFAs expression in fecal matter, while a negative association was observed between blood pressure and the expression of SCFAs-producing microorganisms (Verhaar et al., 2020). The observed outcome can be attributed to the relationship between fecal SCFA levels and the difference between intestinal SCFA production and SCFA absorption. This suggests that an increase in intestinal SCFA expression may lead to an elevated absorption rate of SCFAs (Yang et al., 2019). Moreover, the impact of SCFAs on various receptors can yield diverse regulatory effects on blood pressure. In animal experimentation, it has been observed that the SCFAs receptor Olfr78 in mice plays a significant role in the release of renin, resulting in vasoconstriction and subsequent elevation in blood pressure. Conversely, Olfr78-null mice exhibit diminished levels of plasma renin and consequently experience lower blood pressure (Natarajan et al., 2016). The human counterpart of Olfr78, known as OR51E2, exhibits responsiveness to acetate and propionate, while remaining unresponsive to butyrate. Several receptors for SCFAs are part of the free fatty acid receptor (FFAR) family, such as Gpr41, Gpr43, and Gpr109A. Studies have demonstrated that mice lacking Gpr41 display systolic hypertension, characterized by elevated systolic blood pressure while diastolic blood pressure remains unchanged. This observation suggests that the impact of SCFAs on vasodilation may contribute to this phenomenon (Natarajan et al., 2016; Pluznick, 2017). This implies that Gpr41 plays a beneficial role in the maintenance of blood pressure stability in mice. The contrasting effects of Olfr78 and Gpr41, despite being activated by the same signal, can be attributed to their distinct EC50 levels. Olfr78 exhibits a high EC50 and is activated when SCFA levels are elevated, leading to an elevation in blood pressure. Conversely, Gpr41 possesses a lower EC50 and may experience partial activation even at basal SCFA concentrations, thereby exerting a blood pressure-reducing influence. SCFAs have the ability to modulate blood pressure within a desirable range through the activation of two distinct receptors (Pluznick, 2014). Although recent studies indicate that Gpr43 and Gpr109A also possess antihypertensive properties, the available evidence is limited and the underlying mechanism remains unclear, necessitating further investigation.

TMA is synthesized by intestinal microorganisms via the metabolic breakdown of choline and L-carnitine present in dietary sources. Subsequently, TMA undergoes oxidation by the hepatic enzyme flavin monooxygenase 3 (FMO3), leading to the formation of TMAO (Crothers and Bry, 2018). Recent investigations conducted on animals have indicated that TMAO does not directly induce elevation in blood pressure; however, it can facilitate the augmentation of blood pressure in hypertensive rats (Zhu Y. et al., 2020). There is limited research available regarding the regulatory mechanism of TMAO on blood pressure. Presently, it is hypothesized that TMAO can impact blood pressure by influencing the structure of receptors and angiotensin II (Ang II; Ufnal et al., 2014). Conversely, recent investigations have indicated that TMAO might be implicated in the development of atherosclerosis (Liang et al., 2023). TMAO assumes a significant role in facilitating the transformation of macrophages into foam cells, which are crucial cells involved in the pathogenesis of atherosclerosis (Wang et al., 2019; Zhu Y. et al., 2020). Furthermore, TMAO also serves as a mediator for macrophage migration and augments the expression of inflammatory cytokines (Zhu Y. et al., 2020). A comprehensive elucidation of this particular mechanism has been previously provided by researchers and will not be reiterated herein.

### 4.1 SCFAs

One of the significant metabolites produced by intestinal microorganisms is SCFAs, which have the ability to traverse the BBB and exert their influence on the brain (Mitchell et al., 2011). Recent research has demonstrated that SCFAs possess the capacity to stimulate the release of anti-inflammatory agents while inhibiting the expression of diverse inflammatory factors. For instance, butyrate has been found to selectively diminish the pro-inflammatory response of cytokines such as IL-1β, IL-6, and TNF-α, while simultaneously enhancing the expression of IL-10, thereby mitigating inflammation. Propionate has been shown to have a regulatory impact on the expression of IL-1β and TNF-α, as evidenced by previous research (Sam et al., 2021). The anti-inflammatory effects of SCFAs primarily occur through the TLR4 signaling pathway, distinguishing them from other members of the TLR family (Sam et al., 2021). TLR4, which is predominantly expressed in microglia and astrocytes within the CNS, has also been detected in neurons (Lehnardt et al., 2003; Caso et al., 2007; Lin et al., 2012; Fei et al., 2019). Following ICH, blood-borne substances are discharged into the brain, leading to the activation of microglia through TLR4 receptors via MyD88/TRIF signaling pathway. This activation results in the release of inflammatory signals within the brain, exacerbating the inflammatory response (Lin et al., 2012). Consequently, the modulation and impact of TLR4 receptors by SCFAs exhibit a beneficial influence on the management of brain injury subsequent to ICH. Thus, investigating the potential utilization of SCFAs in the treatment of ICH represents a promising avenue for future research.

#### 4.1.1 Acetate and propionic acid

Upon entering the brain, acetate undergoes conversion into acetyl coenzyme A (acetyl-CoA) through the collaborative activity of acetyl-CoA synthetase 1 (AceCS1; Ariyannur et al., 2010) and AceCS2 (Fujino et al., 2001). Following oral administration of glycerol triacetate (GTA), the acetyl-CoA concentration in the brain exhibits an approximate 2.2-fold increase compared to pre-administration levels. Additionally, this supplementation leads to a reduction in the population of activated microglia and reactive astrocytes (Reisenauer et al., 2011), suggesting that oral acetate acid supplementation may possess anti-inflammatory properties within the brain. Given that acetate primarily affects astrocytes in the brain (Waniewski and Martin, 1998), it follows that astrocytes can influence the activation of microglia (Kim et al., 2010), which serve as key mediators of inflammation. Consequently, the release of pro-inflammatory cytokines, reactive oxygen species, and other substances (Aloisi, 2001) can occur, leading to detrimental effects on the brain. This implies that acetate might impede the communication within these inflammatory cell pathways, thereby exerting a safeguarding influence on brain tissue.

Research has demonstrated that propionic acid exhibits a notable capacity to elevate regulatory T cells in both plasma and feces, while concurrently diminishing pro-inflammatory T helper (Th)1 cells within the brain. Consequently, this mechanism exerts a safeguarding influence on cerebral well-being (Duscha et al., 2020). T cells assume a crucial function in the subsequent neuroinflammatory progression following cerebral hemorrhage (Tschoe et al., 2020). Following the rupture of cerebral blood vessels, M1 microglia cells initiate the release of inflammatory factors, including IL-1β and TNF-α, which contribute to the degradation of extracellular matrix, cellular integrity, and BBB permeability. Additionally, these inflammatory factors recruit and stimulate A1 reactive astrocytes and Th1 differentiation, thereby amplifying the production of inflammatory factors, intensifying the activation of M1 microglia cells, and exacerbating the inflammatory response (Tschoe et al., 2020). Following ICH, there is observable infiltration of intestinal T cells in the hematoma region of the brain. Moreover, the introduction of normal mouse intestinal microorganisms into the intestinal tract of ICH mice has been found to diminish the infiltration of cytotoxic T cells surrounding the hematoma, decrease the expression of inflammatory factors, and enhance the neurological function of ICH mice (Yu et al., 2021). These aforementioned anti-inflammatory effects are potentially mediated by propionic acid.

#### 4.1.2 Butyrate

Butyrate, a frequently encountered SCFA, possesses various functions such as safeguarding the integrity of the BBB, exhibiting anti-inflammatory properties, and preventing apoptosis (Tran and Mohajeri, 2021). The impairment of BBB integrity is recognized as a contributing mechanism to ICH pathogenesis. Recent investigations have demonstrated that both butyrate and propionic acid can enhance BBB integrity, thereby conferring a protective influence on the brain (Braniste et al., 2014; Hoyles et al., 2018). Tight junctions play a vital role in the maintenance of BBB functionality (Zhang et al., 2016). Specifically, claudin-5 and occludin, two significant proteins within tight junctions, are essential for preserving the integrity of the BBB (Förster, 2008). Research has demonstrated that alterations in the expression levels of claudin-5 and occludin can lead to functional changes in the BBB (Zlokovic, 2008). Consequently, it is imperative to uphold the relative stability of both protein expression levels to ensure the continued functionality of the BBB. The preservation of BBB integrity during the initial phase following ICH has been found to have a favorable influence on ICH prognosis (Keep et al., 2014). A study has demonstrated a notable reduction in the expression levels of claudin-5 and occludin in brain tissues of animals post-ICH (Yan et al., 2022). Conversely, oral administration of sodium butyrate to mice has been shown to enhance occludin expression in the brain, consequently leading to a decrease in BBB permeability (Braniste et al., 2014). These findings suggest that butyrate might possess a protective effect on BBB integrity. Currently, there exists no direct empirical evidence supporting the notion that butyrate can directly stimulate the upregulation of claudin-5 expression in the brain. Nevertheless, studies conducted on germ-free mice have revealed a low expression of claudin-5, which subsequently increases following exposure to gut microbiota. This observation indirectly suggests a potential beneficial impact of gut microbiota on claudin-5 expression (Braniste et al., 2014). However, the precise underlying mechanism and the specific substance responsible for this phenomenon necessitate further investigation.

Butyrate has the ability to modulate the expression of pro-inflammatory and anti-inflammatory genes, thereby exerting control over the inflammatory response. Sodium butyrate, functioning as an inhibitor of histone deacetylase (HDAC), can impede the activation of microglia, consequently leading to anti-inflammatory outcomes (Patnala et al., 2017). According to *in vitro* research, sodium butyrate has been observed to modify the enrichment and transcription of histone 3-lysine 9-acetylation (H3K9ac) at the promoter region of genes associated with both pro-inflammatory and anti-inflammatory responses. Additionally, it has been found to enhance the expression of downstream anti-inflammatory factor IL10/STAT3 genes, thereby demonstrating its potential to exert anti-inflammatory effects (Patnala et al., 2017). Following ICH, astrocytes undergo activation, thereby modulating the secretion of diverse pro-inflammatory and anti-inflammatory factors, ultimately leading to inflammation within the brain (Huang et al., 2022). Butyrate has the ability to interact with angiotensin type I receptors, thereby facilitating the regulation of astrocytes and exerting central anti-inflammatory effects (Yang et al., 2018). Monocarboxylate transporter member 1 (MCT1), a thoroughly studied transmembrane protein, actively transports SCFAs and lactate, playing a crucial role in the process of neuroinflammation (Kong et al., 2019). The potential of butyrate to enhance the expression of the MCT1 gene via the NF-κB pathway, leading to the mitigation of inflammation, has been indicated (Borthakur et al., 2008). This implies that butyrate may modulate various genes in order to exert its anti-inflammatory properties, warranting additional research.

Furthermore, butyrate demonstrates dose-dependent anti-apoptotic effects, with low doses typically exhibiting such effects and high doses promoting apoptosis (Zhang et al., 2010). Animal studies have revealed that the supplementation of butyrate significantly enhances the expression of brain-derived neurotrophic factor (BDNF) in the rat brain, consequently downregulating caspase-3 expression and exerting anti-apoptotic effects (Kim et al., 2009). Presently, there exists a scarcity of research concerning the anti-apoptotic impacts of butyrate, with the majority of studies concentrating on the realm of butyrate-induced apoptosis in cancer cells. Neuronal apoptosis plays a pivotal role in the unfavorable prognosis of ICH, with heightened caspase-3 expression serving as a significant indicator. By supplementing with butyrate, the downregulation of caspase-3 expression is observed, thereby demonstrating anti-apoptotic characteristics and suggesting the potential for treating neuronal apoptosis in ICH. Consequently, further comprehensive investigation is warranted in this area.

### 4.2 TMA/TMAO

Furthermore, the metabolic activities of intestinal microorganisms not only generate SCFAs, but also produce TMA, which undergoes oxidation in the liver to form TMAO (Crothers and Bry, 2018). TMAO, being an organic compound capable of traversing the BBB (Del Rio et al., 2017), holds promise as a potential contributor to CNS functions. Nevertheless, the involvement of TMAO in ICH remains a subject of controversy (Zhai et al., 2021; Li C. et al., 2022). Empirical investigations have demonstrated an independent association between TMAO and the 3-month prognosis of ICH patients (Zhai et al., 2021), with TMAO exhibiting a stronger correlation with hemorrhagic stroke compared to ischemic stroke (Nie et al., 2018). However, animal experiments have demonstrated that TMAO has the potential to induce cellular inflammation through the activation of microglia and astrocytes surrounding the hematoma. Nevertheless, it is important to note that this particular effect does not exacerbate brain tissue damage or result in long-term neurological impairments (Li C. et al., 2022).

## 5 Discussion

**Figure 1:**
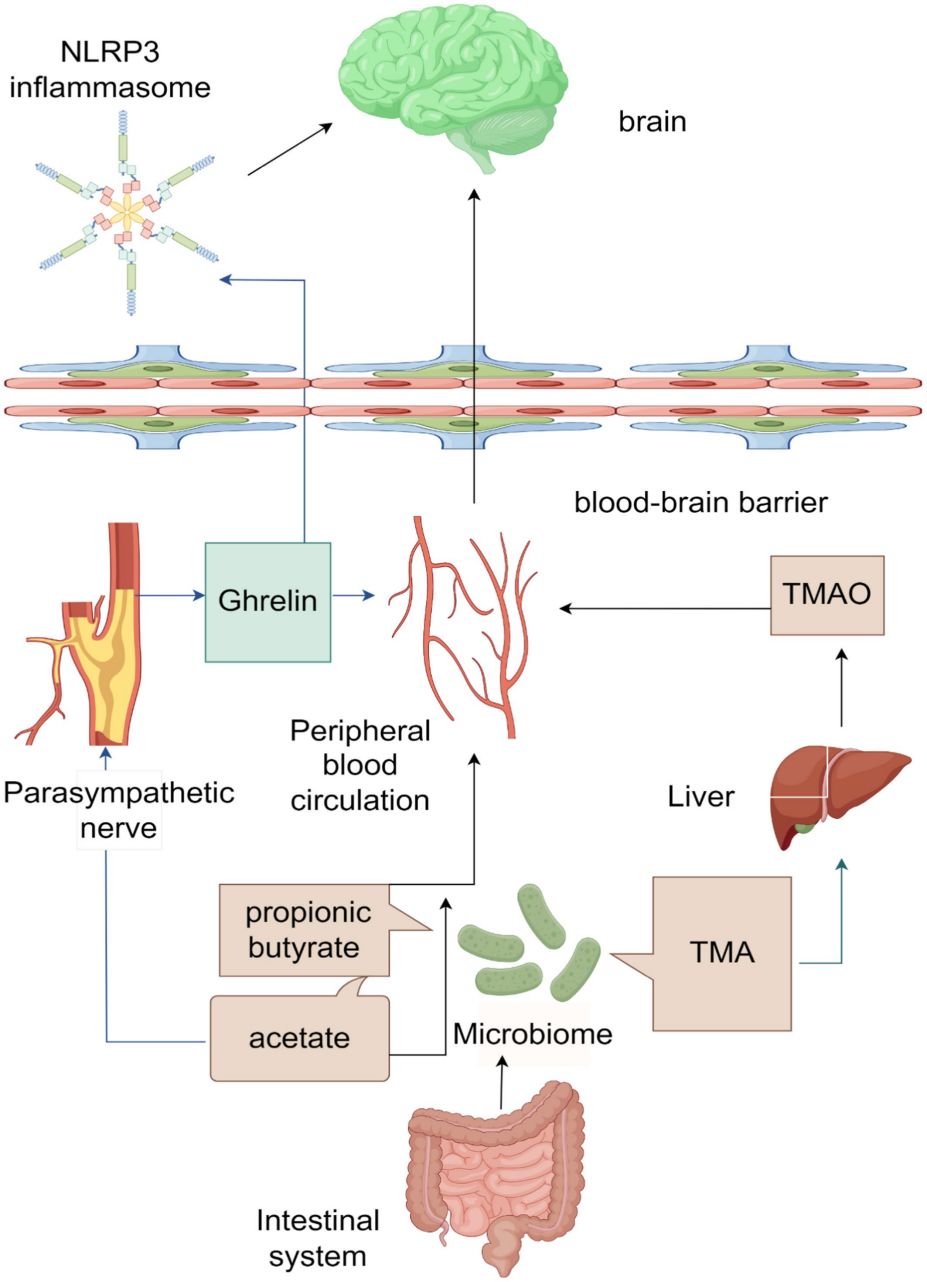
Schematic diagram of the microbiota-gut-brain axis (ICH) acting on the brain after intracerebral hemorrhage (ICH).

The MGBA, a neuroendocrine network of considerable complexity and sophistication, arises from the brain-gut interaction theory. This theory posits the existence of a bidirectional information pathway connecting the brain and the gut, with gut microorganisms and their metabolites playing a crucial role in this pathway, thereby constituting the MGBA. Evidence has demonstrated that the exchange of information and transmission of transmitters between the brain and the gut exert an influence on the onset and progression of specific cerebrovascular diseases, including ischemic stroke (Zhang et al., 2023). However, there is a limited amount of literature available regarding the involvement of MGBA in ICH. Through an examination of pertinent data, this article posits that gut microorganisms and their metabolites, namely SCFAs and TMAO, possess the ability to not only permeate the BBB directly via the peripheral blood, but also exert an influence on the parasympathetic nervous system through the presence of acetate in SCFAs. This, in turn, facilitates the secretion of ghrelin, which can impact the CNS through various pathways, ultimately safeguarding brain tissue (Li et al., 2004; Liu F. et al., 2019; Cheng et al., 2020). A visual representation of this process can be observed in Figure 1.

The main products of the gut microbiota are SCFAs and TMA, among which the main products of SCFAs are acetate, Propionic Acid, and butyrate. SCFAs can directly enter the peripheral blood circulation, cross the blood–brain barrier (BBB), and affect the brain. Acetate can also affect the parasympathetic nerve, promoting the production of ghrelin, which can enter the peripheral blood circulation and act on the brain. Ghrelin can also cross the BBB and affect NLRP3 inflammasome, thereby exerting its effects in the brain. TMA is converted to TMAO in the liver, which then enters the bloodstream and affects the brain.

Hypertension and arteriosclerosis are two commonly recognized factors influencing ICH (An et al., 2017). The metabolites of gut microbiota, SCFAs, can increase blood pressure by binding to Olfr78 and exert a hypotensive effect by binding to Gpr41 receptors, thereby regulating blood pressure (Pluznick, 2014). TMAO has been found to have an indirect impact on blood pressure by exacerbating pre-existing hypertension (Zhu Y. et al., 2020). Furthermore, TMAO has the ability to facilitate the transformation of macrophages into foam cells, thereby contributing to the progression of atherosclerosis (Wang et al., 2019; Zhu Y. et al., 2020). In the future, it may be possible to start from this aspect and regulate the quantity of different SCFAs, TMAO, and receptors in order to control blood pressure and achieve the goal of preventing the onset of ICH.

SCFAs and TMAO not only affect risk factors related to cerebrovascular diseases but also have an impact on ICH. Among them, SCFAs can cross the BBB and exert anti-inflammatory effects in the brain to protect brain tissue (Mitchell et al., 2011). In the human body, the three main components of SCFAs are acetate, propionic, and butyrate. Furthermore, SCFAs and TMAO possess the potential to influence ICH in addition to their impact on risk factors associated with cerebrovascular disease. SCFAs have the ability to traverse the BBB (Mitchell et al., 2011), thereby exerting anti-inflammatory properties within the brain to safeguard cerebral tissue. Acetate, propionic acid, and butyrate constitute the primary constituents of SCFAs within the human body. Butyrate has protective, anti-inflammatory, and anti-apoptotic effects on BBB integrity. Oral supplementation of butyrate can increase the expression level of occludin in the rat brain, enhance tight junctions, and reduce BBB permeability, thereby protecting brain tissue (Braniste et al., 2014). The anti-inflammatory properties of butyrate primarily involve the regulation of pro-inflammatory and anti-inflammatory genes. Butyrate can modulate the enrichment and transcription of promoters associated with these genes, as well as influence the expression of downstream anti-inflammatory genes such as IL-10/STAT3 genes, thereby exerting its anti-inflammatory effects (Patnala et al., 2017). Furthermore, butyrate may also enhance the expression of the MCT1 gene via the NF-κB pathway, leading to increased expression of the MCT1 transmembrane protein and subsequently alleviating neuroinflammation (Borthakur et al., 2008). Presently, existing research indicates that butyrate has the potential to enhance the expression of BDNF, thereby exerting anti-apoptotic effects. However, the precise mechanism by which this occurs and its implications for the prognosis of ICH warrant additional investigation. Similarly, the impact and underlying mechanism of action of TMAO, another microbial metabolite, on brain tissue following ICH remain contentious and necessitate further research.

In addition to directly acting across the BBB to brain tissue, acetate can also act on the parasympathetic nervous system, promoting the secretion of ghrelin and inhibiting TNF-α mediated NF-κB pathway, which inhibits the activation of downstream NLRP3 inflammasomes (Perry et al., 2016; Liu et al., 2022). This ultimately inhibits CNS inflammation and apoptosis processes, thereby protecting brain tissue (Li et al., 2004; Liu F. et al., 2019; Cheng et al., 2020). These findings suggest that ghrelin exhibits remarkable anti-inflammatory properties. Moreover, ghrelin also demonstrates exceptional antioxidant capabilities. According to current research, it has been suggested that ghrelin may potentially exhibit antioxidant properties via the Nrf2/ARE pathway; however, the precise mechanism behind this phenomenon has yet to be thoroughly investigated (Cheng et al., 2020). Furthermore, ghrelin has been found to stimulate the secretion of growth hormone (Fukumori et al., 2012), and a deficiency in growth hormone has been linked to oxidative stress (Kokoszko et al., 2008). Therefore, it is plausible that ghrelin may also exert antioxidant effects by stimulating the secretion of growth hormone. Additionally, ghrelin has been observed to possess anti-apoptotic capabilities within the CNS. Ghrelin has been observed to decrease the Bax/Bcl-2 ratio and suppress caspase-3 expression via the PI3K/Akt and MAPK signaling pathways, thereby exhibiting an anti-apoptotic influence within the brain (Chung et al., 2008; Bai et al., 2022). Apart from its beneficial impact on brain injury subsequent to ICH, ghrelin may also confer protection to the compromised gastrointestinal tract. Nevertheless, additional investigation is necessary to ascertain whether this effect contributes to intestinal injury following ICH.

The NLRP3 inflammasome has been found to facilitate the release of various inflammatory factors, such as IL-1β, within the brain tissue affected by ICH. When the NLRP3 gene is disrupted or the NLRP3 inflammasome is suppressed, the levels of inflammatory factors in the hematoma region of the ICH decrease, leading to a reduction in hematoma size (Ma et al., 2014; Chen et al., 2020). These findings suggest that NLRP3 and the NLRP3 inflammasome play a role in amplifying inflammatory signals in brain tissue following ICH. By inhibiting this process, it is possible to achieve anti-inflammatory effects and safeguard the integrity of brain tissue. Furthermore, apart from its anti-inflammatory properties, NLRP3 also exhibits neuroprotective effects (Xiao et al., 2022). aside from the aforementioned mechanism involving acetate’s impact on the parasympathetic nerve to stimulate ghrelin secretion and subsequently hinder the NLRP3 inflammasome, it appears that the enhancement of the intestinal barrier function by the indigenous gut microbiota also exerts an inhibitory influence on the NLRP3 inflammasome (Zhao et al., 2022). Nevertheless, the precise mechanism behind this phenomenon remains ambiguous and necessitates additional investigation.

This review mainly elaborates on the impact of MGBA on brain tissue after ICH. To elucidate the impact of diverse factors on ICH, Table 1 was constructed to illustrate the varying effects of different authors’ experiments on the pathology. MGBA has been a research hotspot in recent years, but there are still many shortcomings in related research. In recent years, relevant studies have shown that MGBA may have an impact on cerebrovascular diseases (Zhang et al., 2023), but there is relatively little research on ICH. Therefore, there are still many imperfections in this review. Future research can refine the role of MGBA in ICH, such as investigating the prognostic effect of the microbiome on different brain bleeding volumes and bleeding sites, as well as exploring the impact of ICH with varying bleeding volumes on gut microbiota. These areas are all deserving of further research. The potential for intestinal microbiota as probiotics in research is vast. This article delves into the anti-inflammatory, anti-apoptotic, and antioxidant properties of gut microbiota in cases of ICH. These effects have the potential to mitigate brain hematoma and inflammation, as well as provide a protective function for brain tissue. Recent research has also demonstrated the ability of beneficial gut microbiota to maintain gut ecosystem stability (Lee et al., 2020), enhance gut barrier function (Abdelhamid et al., 2022), and potentially impact aging processes (Ros and Carrascosa, 2020). Despite current knowledge, there remains a plethora of undiscovered beneficial functions of gut microbiota in human health, warranting further investigation in future studies. In summary, this article found through research that the microbiome can not only regulate risk factors for ICH, such as blood pressure, but also have an impact on processes such as inflammation and apoptosis. It has great research potential for the treatment of ICH, and there are still many aspects of the microbiome that have not been elucidated in current research. Future research requires further investigation.

**Table 1:** The impact of experiments by different authors on ICH.

	Author(s)	Experimental strategy	Outcome
NLRP3 inflammasome	Shen et al. (2017)	Activated microglia release various inflammatory factors, such as IL-1β.	IL-1β↑
	Ma et al. (2014)	Observing and detecting ICH rats	The earliest increase of IL-1β occurs 3 h after ICH
	Ma et al. (2014)	Inhibiting NLRP3 inflammasome with SiRNA	Decreased brain damage caused by ICH.
	Cheng et al. (2020)	Give calycosin to rats with ICH.	NLRP3 inflammasome↓
			Alleviation of neuroinflammation in ICH rats
	Xiao et al. (2022)	Observation of changes in gut microbiota of ICH rats	High levels of *Helicobacter Pylori* found in the intestines of ICH rats.
	Xiao et al. (2022)	Give MCC950 to rats with ICH.	MBP/NF200↓
Ghrelin	Cheng et al. (2020)	Give ghrelin to rats with ICH and detect inflammatory factors	TNF-α blocks NF-κB pathway, preventing recruitment of inflammatory mediators and NLRP3 inflammasome activation.
	Liu F. et al. (2019)		
	Li et al. (2004)		
	Cheng et al. (2020)	Give ghrelin to rats with ICH	The Nrf2/ARE antioxidant pathway activated
			OS↓
	Bai et al. (2022)	Give ghrelin to rats and detect the central nervous system	The PI3k/Akt and MAPK pathway activated
			Bax↓
			Bcl-2↑
	Chung et al. (2008)		Bax/Bcl-2↓
			Caspase-3↓
	Cheng et al. (2016)	Give ghrelin to rats	CLDN-5↑
			ZO-1↑
Acetate	Reisenauer et al. (2011)	Oral administration of glycerol triacetate (GTA)	Acetyl-CoA↑
			Activated microglia↓
			Reactive astrocytes↓
Propionic acid	Yu et al. (2021)	Observing the effect of Propionic Acid on ICH rats	Cytotoxic T cells around brain tissue hematoma↓
			Inflammatory factor expression↓
Butyrate	Braniste et al. (2014)	Oral administration of sodium butyrate to mice	Occludin↑
			Blood–brain barrier permeability↓
	Yang et al. (2018)	Butyrate interact with angiotensin type I receptors	Regulating astrocytes and exerting anti-inflammatory effects
	Borthakur et al. (2008)	Identification of the butyrate pathway involving NF-κB.	MCT1 gene expression↑
	Zhang et al. (2010)	Effect of different doses of butyrate on apoptosis	Low dose exerts anti apoptotic effect, while high dose promotes apoptosis
	Kim et al. (2009)	Give butyrate to mice and detect the brain	BDNF↑
			Caspase-3↓

## 6 Conclusion

Intestinal microorganisms and their metabolites, such as SCFAs and TMA/TMAO, have the ability to traverse the BBB and influence brain tissue. SCFAs primarily elicit anti-inflammatory responses in brain tissue, whereas butyrate, apart from its anti-inflammatory properties, also exerts anti-apoptotic effects and safeguards the integrity of the BBB. Despite the association between TMAO and ICH, further investigation is required to elucidate its precise function. Additionally, acetate, a type of SCFA, can stimulate the parasympathetic nerve to enhance the secretion of ghrelin. This hormone possesses various beneficial properties, including anti-inflammatory, antioxidant, anti-apoptotic, and neuroprotective effects on brain tissue. Moreover, ghrelin can impede the activation of the NLRP3 inflammasome, thereby safeguarding brain tissue against the buildup of inflammatory factors and exerting anti-inflammatory actions.

## Author contributions

XW: Conceptualization, Data curation, Resources, Writing – original draft, Writing – review & editing. HD: Data curation, Resources, Writing – review & editing. WZ: Supervision, Writing – review & editing.

## Glossary

5-HT: 5-hydroxytryptamine
AceCS1: Acetyl-CoA synthetase 1
Ang II: Angiotensin II
ASC: Apoptosis-associated speck-like protein containing a caspase-recruitment domain
BBB: Blood–brain barrier
BDNF: Brain-derived neurotrophic factor
CNS: Central nervous system
ENS: Enteric nervous system
GHSR: Growth hormone secretagogue receptor
GLDN-5: Claudin-5
GSDMD: Gasdermin D
GSH: Glutathione
GTA: Glycerol triacetate
H3K9ac: Histone 3-lysine 9-acetylation
HDAC: Histone deacetylase
ICC: Interstitial cells of Cajal
ICH: Intracerebral hemorrhage
MBP: Myelin basic protein
MCT1: Monocarboxylate transporter member 1
MGBA: Microbiota-gut-brain axis
NF200: Neurofilament 200
NLRP3: Nod-like receptor 3
PAMP: Pathogen-associated molecular patterns
PRR: Pattern-recognition receptor
SCFA: Short-chain fatty acid
Th: T helper
TMA: Trimethylamine
TMAO: Trimethylamine-N-oxide
ZO-1: Zonula occludens-1
